# Role of emotions in the clinical decision-making process of the hospital nurse: A multicentre qualitative study

**DOI:** 10.1016/j.mex.2024.102590

**Published:** 2024-01-27

**Authors:** Debora Rosa, Giulia Villa, Carla Amigoni, Anna Maria Rossetti, Monica Guberti, Luca Ghirotto, Duilio Fiorenzo Manara

**Affiliations:** a Center for Nursing Research and Innovation, Giulia VILLA, Vita-Salute San Raffaele University, Milan, Italy; b IRCCS Istituto Auxologico Italiano, Milan, Italy; c IRCCS San Raffaele Hospital, Milan, Italy; d Research and EBP Unit, Health Professions Department, Azienda USL-IRCCS Di Reggio Emilia, Reggio Emilia, Italy; e Qualitative Research Unit, Azienda USL-IRCCS di Reggio Emilia, Reggio Emilia, Italy

**Keywords:** Decision making, Emotions, Oncology, Cardiology, Surgery, Outpatient, Rehabilitation, Nursing, The role of emotions in the clinical decision making of hospital nurses: a multicentre qualitative

## Abstract

While for a long-time emotional reaction and moral distress, have been primarily investigated for the possible outcomes of the nursing decision-making process rather than in terms of their role as antecedents of the final decision taken. The primary study's aim is to explore how inpatient nurses' decision-making takes place in different care settings, with a special focus on the role played by emotions during decision-making. The secondary aim is to explore the subjective experience of hospital nurses in relation to successful and unsuccessful decision-making situations.

Multicentre qualitative study, consisting of three phases with different designs: participatory study, grounded theory study, and phenomenological study. Participants will be nurses and may be doctors with various levels of professional experience working in hospital, outpatient, or ward settings. Participants will be recruited through different sampling (purposive and convenience). Data will be collected through focus groups and in-depth interviews with nurses working in different hospital care settings. The researchers expect to find themes that will contribute to a better understanding of the role of emotions in decision-making. The results of this study have the potential of providing important implications to support nurses in the recognition and management of their emotions during the decision-making process.

## Graphical abstract

**Figure:**
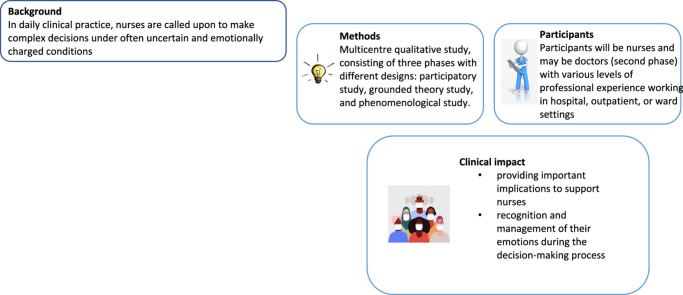

Specifications table

**Table:** 

Subject area:	Environmental Science
More specific subject area:	Nursing Ethics
Name of your protocol:	The role of emotions in the clinical decision making of hospital nurses: a multicentre qualitative
Reagents/tools:	NA
Experimental design:	Qualitative, multi-centric research. Nurses students from different hospitals in Italy will interview. Participants will be recruited through: Phase 1: purposive sampling and maximum variation. Phase 2: the sample will be selected for convenience. Phase 3: the sample will consist of all the participants already identified in the analysis unit for the GT, as well as other participants who meet the inclusion criteria. Data will be collected through focus groups and in-depth interviews with nurses working in different hospital care settings
Trial registration:	NA
Ethics:	The protocol was evaluated and approved by the Local Ethics Committee of the participating institution. The ethical issues relating to studies and research involving human beings set out in the Declaration of Helsinki (2008) have been taken into consideration. All subjects will be informed as fully as possible about all aspects concerning the study. Voluntary participation will be guaranteed. Participants will be informed that non-participation or non-completion of the study will have no negative implications. A written informed consent specific to the study will be obtained prior to participation. The signed and dated informed consent form will be stored in the study file; a copy of the consent will be given to each participating subject.
Value of the Protocol:	- Decision-making is a fundamental and structuring component of everyday clinical nursing practice. - Up to now, most of the studies exploring the role of emotions in nurses' decision-making have been conducted in emergency and urgent departments. - A better awareness of nurses’ experiences is needed. This would help to identify interventions for managing emotions during the decision-making process.

## Description of protocol

### Background

Decision-making is an essential and structuring component of daily clinical nursing practice [1] and refers to the process of judging the nature of care to be provided to patients and how it is delivered [2], [3], [4]. Clinical decision-making can be defined in terms of a discriminative thought process used by the nurse when choosing between alternative options to identify a particular course of action [5,6]. Thus, decision-making represents a contextual, continuous, and dynamic process aimed at gathering information about patients, generating hypotheses about the problem at hand, interpreting data in the light of the suppositions advanced, finding further elements to validate them, identifying the nursing problem and elaborating suitable solutions [7].

A report by Standing of 2008 developed a more comprehensive and complex definition of decision-making in nursing, highlighting the multiplicity of components and knowledge required for effective decision-making, including observation, information processing, critical thinking, problem solving, clinical judgement, ethical values, professional responsibility, science and evidence-based practice [8]. The author underlines how the consideration of all these factors combines theoretical knowledge and practical experience, thus supporting the implementation of behaviours meant to optimize patient's health status while minimising potential harm.

Several interpretative models of decision-making have been previously proposed, most notably the positivist systematic model and the intuitive-humanistic model [9]. The first emphasises the use of explicit analytical processes, traditionally thought to guarantee better outcomes in clinical nursing practice [10]. Large evidence documented how this type of model, in the analytical sequence of its steps, is mostly adopted in the clinical practice by novice nurses [11]. In fact, this approach embraces the deductive hypothetical method and distinguishes four main stages that compose the decision-making process, namely: recognition or acquisition of information, generation of hypotheses, interpretation of information and evaluation of the starting hypotheses [3]. A constant process of data collection and analysis from the particular (the case under investigation) to the general (the acquired knowledge and experience) would lead to the validation or rejection of the originally formulated hypothesis.

On the other hand, the intuitive-humanistic model, that takes its cue from the initial studies by Patricia Benner, identifies intuition as an essential part of clinical judgement. The author emphasises the close link between intuition, experience and the awareness gained from both. Benner and Tanner define intuition as an understanding without a logical foundation and conceptualise it as an art rather than a science [12]. In the view of this model, decision-making would not be linear, logical and analytical scientific reasoning, but the result of rapid, instantaneous, non-logical cognitive processes, mostly unconscious, that derives from intuitive understanding of the situation and practical wisdom acquired through experience. The end product of this process is a tacit, holistic and synthetic knowledge, on which subjects generally rely without having a clear rational justification for their actions [9].

Over time, the view of analytical and intuitive reasoning as two antithetical modes of thinking has been overcome, leading to the formulation of an alternative, more comprehensive and evolved model, which includes both previous approaches as opposite poles of the same cognitive continuum [13]. Based on this notion, the activities involved in decision-making have been then defined through six broad categories referred to two main spheres: the cognitive continuum, which extends from intuition to analysis, and the one relating to the degree of task structuring, which ranges from a low to a high level of structuring [14].

For a long time, emotions have been regarded as a dangerous threat to nursing decision-making and patient safety, to be steered clear of a potential source of judgment bias and errors in clinical reasoning [15].

Advances in neuroscience have led to an increasing revaluation of the traditional view of emotions and cognition as distinct aspects at the brain level [16], contributing to recognize the tight interaction between them within all cognitive processes. Indeed, emotions can be an essential guide in decision-making, significantly influencing the assessment of the salience of a situation, the content of thought, the depth of information processing, and the activation of specific purposes [17].

Emotions play a significant role in decision-making. The effects of emotions are not random or incidental but follow regular mechanisms that influence judgement and decision-making [17]. Therefore, they can have a significant and constant impact on DM, both at a conscious and unconscious level and at an incidental level [18,19]. Conversely, incidental emotions unconsciously and undesirably influence decisions that should not be influenced by that emotion [18,19].

Emotions also influence decisions through various mechanisms, including change in the content of thought, depth of thought, and the content of implicit goals. The positive or negative effects of an emotion on a judgement or decision depend on the interactions between the cognitive and motivational mechanisms triggered by that emotion and the predefined mechanisms that guide decision making. Emotions are not necessarily a form of heuristic thinking. Initially, emotions occur quickly and may lead to an immediate reaction, but once activated, some emotions (such as sadness) may stimulate more systematic thinking [17]. In the context of interpersonal decision making, emotions can serve three main functions: to help individuals understand the emotions, feelings and intentions of others; to incentivise or impose behavioural change on others; and to evoke complementary, reciprocal or shared emotions in others [20,21].

In daily clinical practice, nurses are called upon to make complex decisions frequently under uncertain and emotionally burden some situations, which imply a considerable affective involvement and require the ability to recognise, express, understand, regulate their own and others' emotions and assimilate them into thought processes, to arrive at a constructive clinical judgement [22]. However, only in relatively recent times, nursing literature has started to explore the role assumed by emotions in clinical decision-making; over the past years, instead, emotional reactions and, in particular moral distress, have been mainly investigated in view of the possible outcomes of nursing decision-making rather than for their role as antecedents of the final decision taken [[23], [24], [25]].

Few studies [26] have jointly investigated the emotional and decision-making dimensions, identifying two main themes: the subjective experience of emotions and the application of emotions in decision-making. In line with a previous integrative review on the role of emotional intelligence in nursing practice [27], the authors conclude that subjective emotional experience together with the perception and understanding of others' emotions can exert a powerful impact on clinical decision-making. Other reports [28] have pointed out how the specific emotions experienced and not only their positive or negative valence are the most influential factors on decision-making. It is also emphasised that the basic affective style of healthcare professionals, considered in terms of a dispositional variable, plays a key role on patients’ safety outcomes, both directly and indirectly.

The majority of the studies focused on the effects of emotions in nurses' decision-making have been conducted in clinical settings manifestly characterized by high levels of emotional activation or intense affective involvement, such as emergency rooms and emergency departments [29], intensive care units [30], psychiatry wards [31], outpatient clinics dedicated to voluntary termination of pregnancy [32], and other hospital and non-hospital environments related to palliative care and end-of-life [33,34]. Several investigations have also examined this issue in the oncology setting, however, focusing almost exclusively on advanced clinical conditions requiring transition to palliative or end-of-life care [35].

The decision-making processes that take place in connection with other significant moments of the care pathway, such as diagnosis, definition of a treatment plan, evaluation of alternative treatment options, rejection or abandonment of proposed therapies, etc., have only been analysed by a limited number of studies [36]. Moreover, the available literature does not appear to give a specific consideration on the impact of emotions on decision-making, and often tends to focus primarily on the role assumed by physicians, generally neglecting the contribution of nurses to the decision-making process. The primary study's aim is to explore how inpatient nurses' decision-making takes place in different care settings, with a special focus on the role played by emotions during decision-making. The secondary aim is to explore the subjective experience of hospital nurses in relation to successful and unsuccessful decision-making situations.

## Methodos/ design

This research is a qualitative, multicentre study. The authors wrote the protocol following COnsolidated criteria for REporting Qualitative research (COREQ) [37]. Two hospitals in northern Italy and one in central Italy will participate. The research team chose these hospitals because they have different care settings. They are different from those already studied in the literature.

The protocol is made up of three successive phases, which are interdependent on each other: Phase 1, participatory study; Phase 2, grounded theory; and Phase 3, phenomenological study. In the first two phases, researchers focus on the primary aim. In the third phase, the researchers focus on the secondary aim. Phase one will be the starting point of the study. The other two phases will depend on it. In fact, the care settings from which participants will be recruited for the following phases will be identified during phase one.

For each phase the socio-demographic characteristics of the participants will be analysed by means of descriptive statistics analyses, performed with the IBM Statistical Package for Social Science (SPSS®) statistical software, version 26. Participants’ socio-demographic data in terms of year of birth, gender, profession, length of service, department/ward will be recorded. The NVivo®, software will be used for the management of qualitative data, their analysis, and sharing among the members of the research group.

### Length of study

**Fig. 1:**
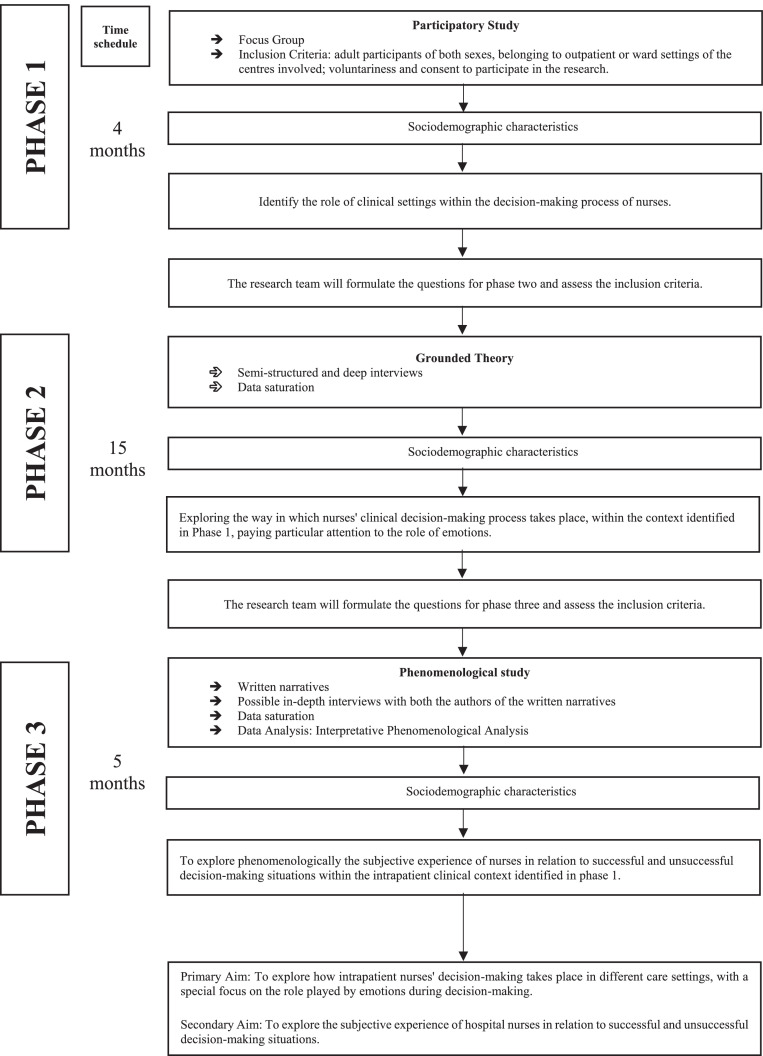
Study Flow.

The study has a total length of 24 months. The first phase will last approximately four months, the second phase approximately 15 months, and the third phase five months (Fig. 1, Study Flow).

### Phase 1: participatory study

#### Participants

Considering the impossibility of defining a priori the entire research sample, but only the selection criteria for the initial sampling, the following types of participants will be considered as eligible for Phase 1: nurses with different degrees of work experience. Inclusion criteria: adult participants of both sexes, belonging to outpatient or ward settings of the centres involved; voluntariness and consent to participate in the research. Exclusion criteria: nurses with reduced knowledge and understanding of the Italian language.

Participants will be recruited through purposive sampling and maximum variation. Each of the three participating centres will define a group of participants (8–12), heterogeneous in terms of gender, seniority, and work context, as previously described [38].

#### Data collection

Data collection will take place through three focus groups. The focus group is a form of group interview that relies on communication between research participants to generate data [38]. Two researchers will conduct the focus groups: a moderator expert in the collection of qualitative data and an observer with the role of descriptor of the non-verbal and para-verbal dynamics between the participants during the interview [39]. Both moderator and observer will be external to the hospital under investigation. Data collection will be audio-recorded and carried out vis-a-vis and/or using a videoconferencing software (Microsoft Teams®, Skype®, Google Meet®, …) in case of impossible in-person data collection. The following socio-demographic data will be recorded on the sample of subjects involved: year of birth, gender, profession, length of service, related service/operational unit.

#### Data analysis

The data will be examined according to the thematic analysis method [40]. This approach involves the following steps: transcription of the verbatim recordings and full reading, subdivision of the transcripts into conversation sequences and definition of the initial labels, combination of the labels to identify the main themes and sub-themes, commenting on the list of the identified themes to ensure internal consistency, description of the main themes, writing of the first result report.

### Phase 2: grounded theory

#### Participants

The initial grounded theory (GT) sample will be selected for convenience. Participants will be nurses and may be doctors with various levels of professional experience working in hospital, outpatient, or ward settings (oncology, surgery, outpatient, rehabilitation, cardiology). Subsequently, further recruitment will take place using theoretical sampling through constant case comparison [41,42]. In this phase, the researchers decided to include doctors. In fact, theoretical sampling requires starting with an initial group of subjects and then gradually expanding it on the basis of stimuli coming from the emerging theory [43]. The initial, theoretical sampling will consist of approximately 30 participants [44,45].

#### Data collection

In the three participating centres, the clinical settings for data collection will be those identified during the Phase 1 of the study through semi-structured interviews. The guiding interview questions will be formulated on the basis of the focus groups of the previous phase and the analysis of existing literature. Data will be collected as described above for Phase 1, guaranteeing the recommended quality criteria [46].

The semi-structured interview has a cognitive purpose with the primary aim of collecting data; it is guided by an interviewer who uses a questioning track [47]. The semi-structured interview comprises a series of questions to be asked without the need for a pre-determined order, leaving room for possible further investigations [48]. For the interview to run smoothly, it will be the interviewer's concern to create a climate based on non-judgmental listening and mutual trust. Therefore, interviews will be conducted by all members of the research team, who have received special training/supervision in this regard, and the location will be chosen by the participants. The presence of an observer may also be envisaged.

As part of the GT study, the interview will last between 30 and 50 min; the audio recording will be transcribed word for word by a member of the research team. All interviewees will be asked for the possibility of a second meeting, in case of necessity to clarify some aspects and discuss the results of the analysis. Furthermore, the context where to conduct this successive interview will be chosen by the participant.

Also, in this phase of the study, some socio-demographic data on the sample of subjects involved will be recorded (year of birth, gender, profession, length of service, department/ward).

#### Data analysis

The data in this phase will be collected and analysed using the GT method, a general research scheme [44,49] that originated from the sociological area inspired by the so-called 'interpretive paradigm'. Consistent with the methodological approach of GT, the research team will analyse the data, contextually to their collection. The data will be initially coded by two researchers in the team to ensure agreement between the coders/researchers. Afterwards, the first analyses will be shared with all the other researchers. By coding data, GT provides a systematic set of strategies for conceptualizing qualitative data. The development of a theory rooted in data, or a theoretical model is possible through the construction of conceptual categories [44,49]. This analytical process comprises three stages: (1) in the initial or open coding texts are indexed [49] through the use of codes or labels that simplify the data and break them down into meaningful simple units; (2) in the second step, focused coding labels are grouped into conceptual categories and concepts or groups of concepts are identified at a higher level of abstraction than the initial coding labels; (3) lastly, theoretical coding is the phase when the explanatory theoretical model on the phenomenon under study is constructed, consistent with the research question formulated in abstract terms, and the relationships between the identified categories are captured.

The purpose of coding is to construct a theory, i.e., a set of plausible relationships between concepts and groups of concepts (categories) [50,51].

### Phase 3: phenomenological study

#### Participants

In this phase of the research, the sample will consist of all the participants already identified in the analysis unit for the GT, as well as other participants who meet the inclusion criteria, according to the methodological indications by Morse [52].

## Data collection

Two data collection strategies may be used in this phase.

(1) Written narratives (description of an experience in written form) from participants meeting the established inclusion criteria, concerning personal experiences of decision-making and their related impact on the care situation.
(2) Possible in-depth interviews with both the authors of the written narratives and other participants meeting the inclusion criteria. The in-depth interview or free interview is a form of open or unstructured questionnaire with a rather low or minimal degree of focus and structuring. The low degree of structuring and the wide margin of freedom granted to the interviewee should not imply that the interviewer might give up the leadership of the interview [53]. For the phenomenological in-depth interview participants will be asked a stimulus question to investigate their subjective experience in relation to situations of success and failure in decision-making within the clinical field identified in Phase 1.

Also in this phase, data will be collected via audio-recording, vis-à-vis meeting and/or by means of videoconferencing tools.

In the context of the phenomenological study, the interview will last between 60 and 75 min and will be audio-recorded and transcribed word for word by a member of the research team; all interviewees will be asked for a successive meeting, if appropriate, and will chose the context to undergo this second interview.

### Data analysis

For this phase, the chosen method is hermeneutically oriented phenomenology, which is the most suitable for understanding the meaning of everyday experiences. In particular, hermeneutic phenomenology and Interpretative Phenomenological Analysis (IPA) allow a detailed exploration of how participants make sense of their personal and social world. For this reason, research involves a detailed examination of the participants' lifeworld [27], because this is the factor that precisely influences the processes of giving meaning to lived experiences. Considering these characteristics, hermeneutically oriented phenomenology is therefore considered to be the most appropriate to meet the secondary objective of the present study. Phenomenological research of the "hermeneutic-interpretive" type, according to Benner, uses the following analytical steps: isolating paradigmatic cases; identifying repetitive themes within and between the cases; selecting illustrative quotations to describe the themes [12].

These steps take the form of a number of operations, where each transcript is read as a 'case' in itself. Some cases immediately emerge as paradigmatic, and the researcher can use them to deepen the participants' themes of experience; re-reading to isolate recurring themes; identification of example quotations to illustrate the themes.

## Ethical considerations

The study will be conducted in accordance with the Declaration of Helsinki and approved by the Ethics Committee of Ospedale San Raffaele (protocol code 22/2022, 13/07/2022). All subjects will be informed as fully as possible about all aspects concerning the study. Voluntary participation will be guaranteed. Participants will be informed that non-participation or non-completion of the study will have no negative implications. Informed consent will be obtained from the participants prior to participation. The signed and dated informed consent form will be stored in the study file; a copy of the consent will be given to each participating subject.

The investigators will perform the study in accordance with this protocol and current Good Clinical Practice. The integrity and privacy of the information will be protected at all times, in line with the General Data Protection Regulation (GDPR) 2016/679 in Italy, and 196/2003 (Personal Data Protection Code, added by the Legislative Decree 101/2018).

The confidentiality of information provided by participants and their anonymity will be warranted. For this reason, participants' names will be changed in focus group transcripts, interviews, and field notes, using pseudonyms or codes. Any identifying characteristics not relevant to the study will be changed. The names of professionals interviewed or quoted will be removed, leaving only their professional role.

Access to the information will also be protected: only research team members will have admittance to data. Information and audio files will be electronically stored, protected during the study and destroyed after its closure. Participants will be informed of the protocols for protecting, storing, accessing, and destroying the information obtained.

## Rigour

Validity refers to the set of verification procedures and strategies used by the investigators during the research process to achieve reliability. Morse et al. [54] defined “verification” as the process of ensuring rigour, validity, and reliability of qualitative research. It is through verification that the criteria, choices, and steps of qualitative research, from the choice of method to the writing of results, are checked, confirmed, and made certain. Since qualitative research is an iterative and recursive process, it is important to have strategies that guarantee congruence between formulation of the research question, method adoption, use of literature, sampling and selection of participants, choice of data collection strategies, and analysis.

The recursive verification of all these steps helps the researcher to understand when to continue, when to stop, when to modify the research process to achieve reliability (which at this point is no longer just the reliability of the results, but of the entire research design), validity and ensure rigour.

The credibility, transferability, reliability and confirmability of participatory and phenomenological research will be ensured to promote the rigour of the study [55]. During the analysis process, researchers will set aside their potential pre-assumptions [27] to focus as much as possible on the participants' experiences [56]. An expert in qualitative studies will supervise the analysis process and two researchers who did not participate in the first analysis will confirm the identified findings [57]. Furthermore, to assess the validity of the GT, the suggested criteria are credibility, originality, resonance and usefulness [50]. Credibility will be achieved by collecting adequate data to support the theoretical explanation of the process. Originality will be achieved by outlining the participants' meanings. As for resonance, saturation will be achieved by obtaining a complete picture of the experience within a specific cultural context. Finally, the research topic will assess the usefulness of this study as it can offer coherent practical implications.

The study will develop thanks to moments of verification with the research team and the scientific supervisor to share the analysis of the data among all researchers and the construction of the results: in this sense, the study has the possibility to measure in itinere the consistency between the coders (intercoder agreement), which is so important to define the rigour and reliability of a qualitative research.

Researchers will also perform the member check before closing the study, especially in the final stages of data analysis. The member check is the activity of going back to the respondents and asking them whether they agree with what the researcher has interpreted, and it is not a verification strategy [49], but a way of making respondents sensitive to a particular issue

## Discussion

The present study protocol was developed to explore how inpatient nurses' decision-making takes place in different care settings, with a special focus on the role played by emotions during decision-making, and to explore the subjective experience of hospital nurses in relation to successful and unsuccessful decision-making situations.

Decision-making critically affects the quality of patients’ care, safety and satisfaction, as well as multiple clinical outcomes [58]. Within traditional interpretative models, despite their progressive evolution, individual emotions do not seem to have gained adequate recognition or being invested with a prominent function and value in clinical decision-making [26]. In view of the crucial role of emotions in responding to environmental stimuli through their ability to activate and coordinate a variety of reactions at physiological, behavioural and experiential levels, the multiplicity of functions they assume in modulating decision-making is now widely documented [17].

The results of this study have the potential to give contribution for the development of a theory that helps to explain the role of emotions in everyday clinical nursing practice (analysing care needs, identifying goals, selecting interventions, evaluating effectiveness of care). This would allow researchers to identify which types of decisions are the most critical. Thus, hypotheses formulated on possible supportive interventions could be put in place to help nurses recognise and manage their emotions. Such supportive interventions could also be structured in training and awareness-raising initiatives for staff. Furthermore, it could be useful to create tools for assessing the impact of emotions, which would enable healthcare organisations to better understand the problem, to implement the most appropriate interventions and to monitor the situation over time or in case of emergencies.

## Limitations

The identification of the care setting where to conduct the second phase of the study could be influenced by the subjects who will constitute the focus groups. Moreover, the Italian experience during the COVID-19 pandemic may have changed and broadened the perception of emotions during the decision-making process, especially since this challenging situation has not yet been fully processed by all healthcare professionals [59]. A third limitation might be the poorly available literature dealing with this phenomenon, which might prevent us to carry out fitting comparative evaluations with similar previous experiences on the same matter.

The development of the protocol could contribute to professionals' awareness of the presence of emotions during the daily care of patients. Furthermore, the knowledge gained through this project could be useful in identifying organisational strategies that enable the knowledge and management of emotions. The expected results of this protocol include the classification of care settings in which emotions play an important role during decision-making.

## CRediT authorship contribution statement

**Debora Rosa:** Methodology, Investigation, Data curation, Formal analysis, Writing – original draft. **Giulia Villa:** Conceptualization, Investigation, Methodology, Writing – original draft, Funding acquisition. **Carla Amigoni:** Writing – review & editing. **Anna Maria Rossetti:** Writing – review & editing. **Monica Guberti:** Writing – review & editing. **Luca Ghirotto:** Methodology, Formal analysis, Writing – review & editing, Supervision. **Duilio Fiorenzo Manara:** Conceptualization, Methodology, Investigation, Funding acquisition, Supervision.

## Declaration of competing interest

The authors declare that they have no known competing financial interests or personal relationships that could have appeared to influence the work reported in this paper.

## Data Availability

No data was used for the research described in the article.
